# Sex-Divergent Clinical Outcomes and Precision Medicine: An Important New Role for Institutional Review Boards and Research Ethics Committees

**DOI:** 10.3389/fphar.2017.00488

**Published:** 2017-07-21

**Authors:** Ignacio Segarra, Pilar Modamio, Cecilia Fernández, Eduardo L. Mariño

**Affiliations:** Clinical Pharmacy and Pharmacotherapy Unit, Department of Pharmacy and Pharmaceutical Technology and Physical Chemistry, Faculty of Pharmacy and Food Sciences, University of Barcelona Barcelona, Spain

**Keywords:** sunitinib, sex-divergent pharmacokinetics, clinical outcomes, covariate sex, research ethics, IRB, ELSI

## Abstract

The efforts toward individualized medicine have constantly increased in an attempt to improve treatment options. These efforts have led to the development of small molecules which target specific molecular pathways involved in cancer progression. We have reviewed preclinical studies of sunitinib that incorporate sex as a covariate to explore possible sex-based differences in pharmacokinetics and drug–drug interactions (DDI) to attempt a relationship with published clinical outputs. We observed that covariate sex is lacking in most clinical outcome reports and suggest a series of ethic-based proposals to improve research activities and identify relevant different sex outcomes. We propose a deeper integration of preclinical, clinical, and translational research addressing statistical and clinical significance jointly; to embed specific sex-divergent endpoints to evaluate possible gender differences objectively during all stages of research; to pay greater attention to sex-divergent outcomes in polypharmacy scenarios, DDI and bioequivalence studies; the clear reporting of preclinical and clinical findings regarding sex-divergent outcomes; as well as to encourage the active role of scientists and the pharmaceutical industry to foster a new scientific culture through their research programs, practice, and participation in editorial boards and Institutional Ethics Review Boards (IRBs) and Research Ethics Committees (RECs). We establish the IRB/REC as the centerpiece for the implementation of these proposals. We suggest the expansion of its competence to follow up clinical trials to ensure that sex differences are addressed and recognized; to engage in data monitoring committees to improve clinical research cooperation and ethically address those potential clinical outcome differences between male and female patients to analyze their social and clinical implications in research and healthcare policies.

## Introduction

The research drive toward personalized medicine has grown exponentially during the last years in an attempt to improve therapeutic outcomes putting the patient at the center of the healthcare system. The shift toward precision medicine has deeply focused on diagnosis and treatment, particularly in the oncology field (Prasad et al., 2016). The development of small molecules to inhibit specific molecular pathways in oncology led to pioneer approval in 2001 of imatinib, a signal transduction inhibitor targeting TK cKIT, PDGFα/β, and Bcr-Abl receptors for the treatment of GIST (Dagher et al., 2002). Following imatinib, a series of other TK inhibitors were introduced and approved providing new therapeutic options for a variety of cancers (Wu et al., 2016). Simultaneously, further personalized strategies for patient treatment combining TDM and dose individualization were being developed (de Wit et al., 2015; Stinchcombe, 2017).

**FIGURE 1:**
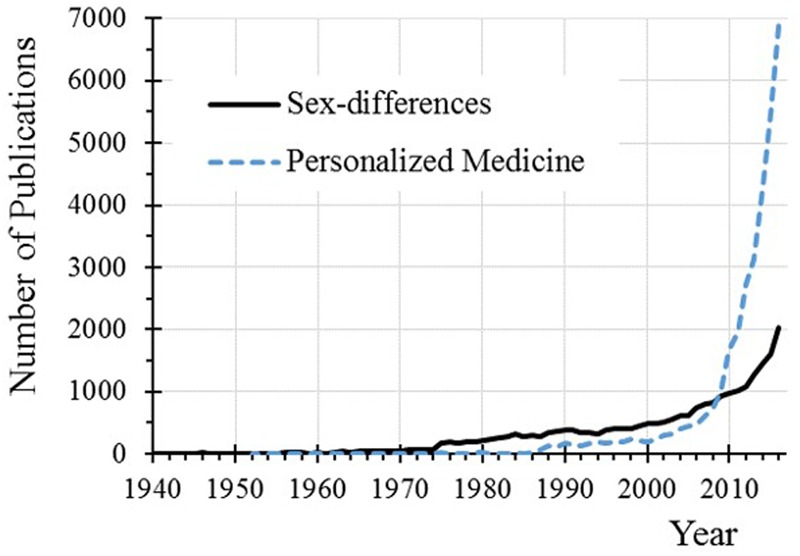
Number of publications in PubMed dealing with sex-divergent and personalized medicine. The searching terms were “personalized medicine” and [“sex-differences” OR “sex-divergent” OR “sex-dependent”] for sex-differences.

The emphases on personalized treatment to improve individual therapeutic outcomes has encouraged the identification of clinical outcomes differences between male and female patients (**Figure 1**). Based on a deeper understanding of their nature, sex differences have been grouped in four types (Becker et al., 2017): (1) qualitative sex differences where male and female sexes show different behavior; (2) quantitative sex differences when there is greater response in one of the sexes; (3) convergent sex differences in which both sexes display the same behavior but the pathways that mediate it are different, and lastly (4) population sex differences where the presence of a specific behavior differs in proportion between males and females. The need to provide a deeper understanding and relevance of these differences has moved regulatory and scientific agencies to issued recommendations encouraging the study of their impact on therapeutic outcomes (Bren, 2005). Although the overall implementation has been scarce (Fisher and Ronald, 2010) for different practical reasons (Bolon, 2010; Fisher and Ronald, 2010; Cahill, 2014), there have been various reports addressing potential sex-based differences in drug efficacy and toxicity, at least from a descriptive point of view (Ostrom et al., 2014; Sun et al., 2015). In a large review of clinical studies, differences in efficacy were reported in 1 out of 68 drugs for 36 indications (Gartlehner et al., 2010). However, the authors remarked that *“most available evidence was compromised by methodological limitations”* precluding any deep inquiry about the impact of sex differences on efficacy and toxicity. Further cumulative evidence has matured in various fields (Klein et al., 2015) including neurosciences (Cahill, 2006, 2017), neurodegenerative diseases (Bove and Chitnis, 2013; Canevelli et al., 2017), medication use and treatment adherence (Chen et al., 2014; Manteuffel et al., 2014), incidence of DDI (Bowlin et al., 2013) as well as clinical and preclinical research (Franconi and Campesi, 2014a,b; Mazure, 2016; Segarra et al., 2016). This evidence has pushed forward more clearly the need to address potential sex-based differential clinical effects on male and female patients with the inclusion of covariate sex in the outcome analysis for the benefit of patients (Segarra et al., 2016; Cahill, 2017).

### Tyrosine Kinase Inhibitor Sunitinib

Tyrosine kinase receptors are a subset of protein kinases involved in numerous cellular processes (Roskoski, 2015). Because of their extent and large cell functionality and involvement in pathogenesis and disease progression pathways, intensive efforts to identify and validate therapeutic targets have taken place, leading to the subsequent development of molecules targeting specific TK receptors (Chow and Eckhardt, 2007; Goueli, 2017) which have delivered significant improved clinical outcomes (Wu et al., 2015, 2016).

Sunitinib is a small molecule, able to inhibit a multiplicity of TK receptors including the platelet-derived growth factors PDGFRα/β, the colony stimulating factor type I (CSF-1R), the vascular endothelium growth factor (VEGFR), the stem cell factor c-KIT, and the fetal liver TK receptor 3 (FLT3) amongst others (Faivre et al., 2006; Papaetis and Syrigos, 2009). Owing to this ability to inhibit diverse multiple TK receptors, sunitinib has shown capacity to arrest angiogenesis, metastasis and tumor progression (van Erp et al., 2009). These features have led to its therapeutic application in RCC (Motzer et al., 2016), metastatic RCC (Schmid and Gore, 2016) including brain metastasis (Lombardi et al., 2014), GIST resistant to imatinib, metastatic GIST (Khosravan et al., 2016), and pancreatic neuroendocrine tumors (Blumenthal et al., 2012; Capozzi et al., 2016).

We have carried out the analysis of the translational impact of covariate sex on an anticancer drug such as sunitinib and compiled various ethic proposals to better address the impact of sex on clinical outcomes and trigger scientific discussion to improve the design of future research.

## Translational Approach of Covariate Sex on Sunitinib

The translational relevance and clinical interest of covariate sex applied to sunitinib treatment has been assessed at different levels: (1) the possible sex-divergent pharmacokinetics of sunitinib and (2) the differential effect of DDI on male and female sexes.

### Covariate Sex Effect on Pharmacokinetics

The effect of sex differences on the pharmacokinetics and tissue distribution of TK inhibitor sunitinib was evaluated in a preclinical mouse model (Lau et al., 2015; Liew et al., 2017). In both studies, statistically significant differences between sunitinib exposure in male and female mice were found in plasma, liver, brain, and kidney tissue. This finding may have significant clinical relevance owing the therapeutic use of sunitinib against brain tumors, RCC, and metastatic RCC, two target tissues (brain and kidneys) that showed marked differences between sexes: female mice show higher sunitinib penetration and exposure in brain tissue versus male mice while male mice have greater sunitinib penetration in kidneys *versus* female mice (**Table 1**).

**Table 1:** Effect of DDI on plasma and tissue AUC_0→∞_ (*versus* control groups) after a single dose (60 mg/kg) administration of sunitinib orally.

GROUP	PLASMA		LIVER		KIDNEY		BRAIN	
	Male	Female	Male	Female	Male	Female	Male	Female
*Control ICR*		**>^+++^**	**>^+++^**		**>^+++^**			**>^+++^**
Diclofenac^1^	**↓**^∗∗∗^	**↔**	**↓**^∗∗∗^	**↑**^∗∗∗^	**↓**^∗∗∗^	**↑**^∗∗∗^	**↓**^∗∗∗^	**↓**^∗∗∗^
Paracetamol^2^	**↓**^∗^	**↔**	**↓**^∗∗∗^	**↓**^∗∗∗^	**↓**^∗∗∗^	**↓**^∗∗∗^	**↓**^∗∗∗^	**↓**^∗∗∗^
*Control Balb/c*	**>^+^**			**>^+++^**	**>^+++^**			**>^+++^**
Ibuprofen^3^	**↔**	**↓**^∗∗^	**↑**^∗∗∗^	**↓**^∗∗∗^	**↑**^∗∗∗^	**↓**^∗∗∗^	**↑**^∗∗∗^	**↓**^∗∗∗^

*^+^Significant differences between genders in the control groups: ^+^*p* < 0.05, ^+++^*p* < 0.001. ^∗^Significant differences with the respective control group: ^∗^*p* < 0.05, ^∗∗^*p* < 0.01, ^∗∗∗^*p* < 0.001. ^1^Adapted from Chew et al. (2017). ^2^Adapted from Liew et al. (2017). ^3^Adapted from Lau et al. (2015). DDI studies with diclofenac and paracetamol were carried out in ICR mice and the DDI study with ibuprofen in Balb/c mice. Control ICR and Balb/c are also included showing whether male or female mice attained greater AUC_*0*→∞_.*

An exploratory attempt to correlate these findings with clinical outcomes in male and female patients was previously done, although it was limited to individual cases and case series reports of patients treated with sunitinib (Segarra et al., 2016). The individual cases seemed to suggest that female patients achieved better response to brain tumor than male patients which was concordant with literature observations showing better outcomes and lower incidence of brain tumor in female patients (Sun et al., 2015). In addition, apparent better RCC therapeutic outcomes were observed in male patients (Houk et al., 2009; Bamias et al., 2013). An additional observation was the higher incidence of adverse effects, including various fatal liver failure cases in female patients (van der Veldt et al., 2008; Akaza et al., 2015; Narjoz et al., 2015).

Last, sex-divergent differences in clearance and volume of distribution parameters between male and female patients have been shown (Khosravan et al., 2016) which suggests clinical translatability from preclinical studies. The pharmacokinetic differences between male and female sexes may be responsible for the large variability and lack of efficacy encountered in some clinical trials which led to the change or discontinuation of treatment (Lankheet et al., 2014b; Akaza et al., 2015; Gore et al., 2015; Barrios et al., 2016; Domagała-Haduch et al., 2016) likely due to poor dosing adjustment since plasma concentration alone may not ensure precise dose adjustment (Houk et al., 2010; Lankheet et al., 2014b) as seen in most TK inhibitors (de Wit et al., 2015; Kotecki and Penel, 2016). This is especially relevant if sex differences lead to tissue exposure differences as observed in the preclinical studies, are not taken into account. The translatability interpretation of the preclinical data shows higher sunitinib plasma exposure and uptake in brain in female mice and higher sunitinib penetration in kidney tissue in male mice which may anticipate the clinical differences observed in patients (Segarra et al., 2016).

### Effect on DDI Outcomes

The covariate sex may also have a significant impact on DDI outcomes given the fact of cancer patient polymedication (Leblanc et al., 2015) including those undergoing sunitinib treatment (Bilbao-Meseguer et al., 2015). Sex-based DDI outcome differences were observed upon coadministration of sunitinib with selected NSAIDs, used to reduce cancer pain or side effects related to the treatment (Ripamonti et al., 2014; Hammer et al., 2016; Mercadante and Portenoy, 2016). In preclinical studies, dramatic sunitinib plasma and tissue exposure effects were observed after coadministration with ibuprofen (Lau et al., 2015), paracetamol (Liew et al., 2017) or diclofenac (Chew et al., 2017). These effects included changes of sunitinib plasma exposure and most importantly, changes in brain, liver, and kidney sunitinib penetration that were different in male and female mice showing sex-different plasma-tissue DDI outcomes (**Table 1**): diclofenac and paracetamol decreased plasma exposure in male mice which compares with a general reduction of sunitinib exposure in tissues of interest. However, female mice did not show any DDI effect in plasma and sunitinib exposure remained unchanged eventhough large effects did take place in tissues. Last, ibuprofen showed the opposite behavior: no effect was observed in sunitinib plasma exposure in male mice while significant changes took place in tissues. Similarly, after multiple dose administration to mice (Tan et al., 2016), sex-divergent concentration changes were also observed in plasma and tissues, although statistical significance was attained only upon sunitinib coadministration with paracetamol or ibuprofen (**Table 2**).

**Table 2:** Trend effect of DDI on plasma and tissue concentrations (*versus* control groups) after multiple dose administration of sunitinib orally in ICR mice.

GROUP	PLASMA		LIVER		KIDNEY		BRAIN	
	Male	Female	Male	Female	Male	Female	Male	Female
*Control ICR*		>	>		>			>
Diclofenac	**↓**	**↓**	**↓**	**↔**	**↓**	**↓**	**↑**	**↓**
Paracetamol	**↓**	**↑**	**↑**^∗^	**↓**	**↑**^∗^	**↓**	**↑**	**↓**
Ibuprofen	**↑**	**↓**^∗^	**↑**^∗∗^	**↓**	**↑**	**↓**	**↑**	**↓**^∗∗∗^
Mefenamic Acid	**↑**	**↓**	**↑**	**↑**	**↓**	**↓**	**↔**	**↓**

*^∗^Significant differences with the respective control group: ^∗^*p* < 0.05, ^∗∗^*p* < 0.01, ^∗∗∗^*p* < 0.001. Adapted from Tan et al. (2016).*

Likewise, clinical observations identified numerous DDI with sunitinib (Bilbao-Meseguer et al., 2015), including DDI which showed differential effects on male and female patients (Bowlin et al., 2013): female patients showed significantly higher frequency of DDI causing increased toxicity related to the target enzyme. This higher rate of DDI was observed also with other TK receptor inhibitors, imatinib, and erlotinib (Bowlin et al., 2013), making necessary to evaluate appropriately the selection of pain management drugs in cancer patients.

### Translatability in Therapeutic Drug Monitoring

There is an often forgotten translatability issue: TDM. Usually, TDM protocols are developed during the clinical stage and focused on a surrogate parameter (marker) that indicates treatment efficacy or toxicity. Generally, these surrogate parameters are either drug or biomarker concentrations in a measurable matrix (e.g., plasma, blood or urine) or genotyping able to relate to efficacy or toxicity (Eliasson et al., 2013).

The relevance of addressing sex as a covariate strikes hard at the current pharmacotherapy practice and management of TK inhibitors (Lankheet et al., 2014a,b) owing to the polypharmacy scenario of cancer patients (Leblanc et al., 2015): several preclinical DDI studies of sunitinib with selected NSAIDs, have shown that the DDI effects on plasma and tissue were different in male and female mice. Particularly relevant for TDM were the observations that paracetamol and diclofenac caused a reduction of sunitinib plasma exposure in male mice but not in female mice. However, ibuprofen reduced sunitinib exposure in female mice. In all cases, the reduction of sunitinib plasma exposure was accompanied with a subsequent reduction in tissue penetration (**Table 1**). Thus, plasma concentration based TDM would probably detect a DDI with paracetamol or diclofenac in a male patient and a possible DDI with ibuprofen in a female patient. Then, subsequent dosage adjustment may be carried out. However, it is likely that the DDI would remain undetected in female patients taking paracetamol or diclofenac and in male patients taking ibuprofen. In this case, their coadministration would mask changes in brain, liver or kidney sunitinib penetration and exposure as these do not correlate with the events occurring in plasma (**Table 1**). Thus, if monitored parameters remain unchanged in plasma (e.g., plasma concentrations), or cannot be measured in tissues, then, understanding male-female DDI differential effects becomes essential to carry out TDM successfully (Gao et al., 2012; Takasaki et al., 2017). Else, there exists a risk to diminish treatment efficacy or even to treatment failure (e.g., in brain tumor and RCC female patients with concomitant use of paracetamol) or to increase toxicity due to higher liver sunitinib exposure upon coadministration with diclofenac or paracetamol in female patients or with ibuprofen in male patients.

Furthermore, the routine introduction of biomarkers in TDM is an intense research area (Eliasson et al., 2013). This would ensure that TDM captures possible sex-divergent expression of sunitinib clinical outcomes to evaluate the response, the efficacy and the toxicity in male or female patients (Planchard et al., 2009; Wendler and Wehling, 2010; Moon et al., 2013). This ability to integrate covariate sex effects in preclinical data with clinical development outputs may anticipate the goodness, suitability and reliability of sunitinib TDM protocols to anticipate and detect differential outcomes between male and female patients (Franconi and Campesi, 2014a,b).

## Role of Sex in Future Drug Development Clinical Research

The analysis of sunitinib therapeutic outputs in male and female patients, although limited, allows the identification of various domains which are relevant to sex-based clinical research: the introduction of sex as a covariate in the analysis, the integrative translational research approach, the effects on pharmacotherapy management and patient involvement amongst other domains. All these efforts would lead to greater health equity between male and female patients (Chapman, 2010).

### Contextualizing Covariate Sex

The specific clinical importance of sex differences has increased scientific awareness to study them in a systematic manner, particularly in clinical research (Mazure and Jones, 2015). However, as it has been reported recently, their impact on pharmacokinetics and therapeutics has been considered of relatively low importance and in general, male sex has been used as a proxy for female sex (Brooks and Clayton, 2017; Cahill and Hall, 2017). This approach has generated a vacuum of high quality evidence that would have allowed a univocal understanding of the potential influence of sex differences on the pathogenesis, disease progression and in the last analysis, the therapeutic outcomes (Picillo et al., 2017). The quest for precision and personalized medicine has been *“deeply harmful, in particular to the health of women”* (Cahill, 2017) as this information is lacking for developing adequate therapeutic protocols. This possible scenario may be observed with the clinical use of sunitinib to treat brain tumors, RCC and mRCC as well as with the incidence of adverse effects. The reports of the main sunitinib clinical trials differentiate between male and female patients in the demographic data section but fail to report the efficacy and toxicity outcomes differentiating between male and female patients (Segarra et al., 2016). This lack of knowledge may create a gap that could lead, for example, to the coadministration of a less suitable pain management drug (e.g., paracetamol) to a brain tumor female patient. In turn, this may lead to diminish the drug efficacy, more over without TDM revealing any change of the plasma concentration of sunitinib, and thus possibly remaining undetected.

Higher quality evidence addressing possible sex differences impact on therapeutics is lacking. This makes difficult to identify the best and more appropriate pharmacotherapy approaches. However, new opportunities to bridge the gap between male and female patients are emerging upon the paradigm shift taking place in the drug discovery and development process. In fact, some of the differences between sexes were found the hard way, that is to say, once the drug is available to a large public and clinical response differences begin to surface (Cahill and Hall, 2017). Thus, the current shift from a linear drug development process or *“bench to bedside,”* where molecules overcome specific cut-off values to progress to the next stage in a funnel-like competition, toward a translational model (“*bedside to bench”*) is allowing the identification of patient oriented selection strategies (Chorghade et al., 2017). Thus the *“bedside to bench”* approach *versus* the *“bench to bedside”* traditional approach takes advantage of the large number of patients which have already used the drug to identify new therapeutic indications or at risk populations. These findings are followed by sets of preclinical and paraclinical studies which incorporate the novel features identified in the clinical trials and safety studies that brought forward the potential new indication (Lieu et al., 2013). This approach may identify differences in clinical outcomes between male-female patients to conduct treatment adjustment based on each patient sex and enhance further patient oriented research (Fisher and Ronald, 2010).

### Translational Preclinical Research

The life cycle of a drug molecule described above is no longer linear with the pharmaceutical companies increasingly looking for additional therapeutic applications of their marketed products, expanding their therapeutic potential as well as maximizing their financial and scientific investments (Fisher and Ronald, 2010). Cancer therapeutics has successfully benefit from this *“bedside-to-bench-and-back-to-bedside”* approach. Several marketed molecules targeting drivers of oncogenic processes have increased their target repertoire adding other cancer types or specific patient populations that could benefit from them (Lieu et al., 2013): succinct empirical observations gathered from everyday clinical practice were revisited in preclinical models to identify and understand their underlying mechanisms. Tyrosine kinase inhibitor sunitinib illustrates well how preclinical studies carried out post-marketing offered improved treatments (Carlisle et al., 2016).

Various aspects are needed to ensure validity of this translational approach to identify sex-based differences. At cellular level, the sex origin of the cell lines affects the pharmacological outcome (Nunes et al., 2014) owing to different internal cellular mechanisms (Sun et al., 2015) or different expression levels of targeted receptors, conditioned by the male-female genetic makeup of the cell (Hägerstrand et al., 2006). Cellular sex-based differences have a clear and decisive effect on the possible sex-divergent pharmacokinetics of sunitinib: First, intracellular drug metabolizing enzymes are involved in the biotransformation (Sakuma et al., 2009; Waxman and Holloway, 2009) and generation of active metabolites (e.g., sunitinib’s demethyl metabolite). Second, sex-divergent membrane transporters amount and localization (Cui et al., 2009; Hou et al., 2014; Joseph et al., 2015) may restrict tissue penetration and active excretion processes differently (Bebawy and Chetty, 2009; Franconi and Campesi, 2014b; Mazure, 2016). Furthermore, the study of the effects in both sexes should include studies evaluating the whole lifespan in preclinical animal models, to study the impact of sex hormones at different ages, as they have been linked to clinical outcome differences in humans (Gur and Gur, 2016).

Eventhough sex differences between male and female subjects are rooted at the cellular level (e.g., chromosomal determination), they affect the therapeutic response of the subject (Marazziti et al., 2013; Naidoo et al., 2014; Sun et al., 2015). These divergent outcomes demand specific *in vitro* and *in vivo* study designs able to assess the impact of each singular difference upon which particular emphasis is required for P450 enzymes and membrane transporters (Cummins et al., 2002). Therefore, to fully identify the impact of sex on therapeutics seems necessary to develop post-marketing preclinical studies with a clinical outlook taking into account diverse clinical scenarios (Chorghade et al., 2017). Amongst these scenarios, it is crucial to include polypharmacy situations to anticipate adverse events due to the coadministration of various drugs (Zhang et al., 2011). Because female patients are more susceptible to medication use (Bowlin et al., 2013), the analysis of potential DDI in animal models may provide valuable information about the impact and translatability on the efficacy and toxicity to optimize therapy in female patients (Kotecki and Penel, 2016; Peck, 2016) or even to infer novel therapeutic approaches (Lim et al., 2010; Chew et al., 2012; Tan et al., 2013, 2016). In addition, these studies should complement other preclinical studies and support the efforts to identify sex-divergent outcomes (e.g., pharmacokinetics and tissue distribution) and translate the findings to clinically viable options with a better understanding of disease progression, lower attrition rate and improved predictability from basic research (Maienschein et al., 2008).

### The Meaning of Sex as a Covariate

The significance of the covariate sex in crucial to identify possible differences. Women have gradually increased participation in clinical trials (Pinnow et al., 2009) and their participation remains essential to assess whether sex-based differential outcomes exist. If differences do not exist, either population (male or female patients) would provide reliable results regarding the performance of the drug. But this premise cannot be assumed lightly and it needs to be proven in clinical trials that include male and female patients, taking into account the higher risks that female patients may endure (e.g., risk of unknown teratogenic effects).

The evaluation of sex-divergent therapeutic outcomes requires an interpretation beyond the dichotomous cut-off probability *p*-value, e.g., *p* < 0.05 (Wasserstein and Lazar, 2016). Otherwise, a mismatch may develop between the objective endpoints (the effect of sex on the specific measurable clinical outcome) and the statistical criteria limited by the sample size and measurement precision used to calculate the *p*-value (van Rijn et al., 2017). To overcome this risk, it is crucial to report the *p*-value of the results always, regardless whether it is higher, lower or equal to 0.05: this reflects the degree of influence of the covariate on the feature which is being evaluated (Kyriacou, 2016). Furthermore, first, it allows an evidence-based approach to address the impact of sex differences on therapeutic outcomes of male *versus* female patients and, second a holistic approach with a continuum scope linking clinical practice and patient behavior. This approach may ensure whether a non-significant or a significant *p*-value (based on a pre-established *p*-value) is clinically relevant. Factors such as higher medication use including non-prescribed and self-medication, comorbidities and age differences in female patient population (Bren, 2005; Regitz-Zagrosek, 2014) are aspects that may influence the pharmacotherapy outcome and make covariate sex clinically significant. Other aspects related to patient behavior such as lesser access to healthcare and lack of adherence in female patients (Chen et al., 2014; Franconi and Campesi, 2014b; Manteuffel et al., 2014) may also lead to unexpected differential outcomes between male and female patients.

The first steps toward precision medicine appeal to the principal investigators (PI), scientists and study coordinators for implementation of sex-differentiated data analysis in the results section of the study report, regardless of the *p*-value achieved. This action seems essential to evaluate the impact of covariate sex on therapeutics, to assess whether these differences exist and how deeply they modulate clinical outcomes. Establishing this link between the *p*-value and its clinical significance (e.g., differences observed in clinical practice, in patient care and behavior) can indicate whether differential pharmacotherapy approaches in male and female patients are needed. If sex difference impact is inferred, then therapeutics may be adjusted and precision medicine realized (de Wit et al., 2015).

We would greatly encourage researchers and authors to implement sex-differentiated reporting of efficacy, toxicity and endpoint attainment, as well as editors to require this analysis in their scientific publications (Blaustein, 2012; Prager, 2017; Rippon et al., 2017). Furthermore, comparison of results between sexes in upcoming studies, including bioequivalence studies, adaptive clinical trial designs (Lieu et al., 2013), as well as revision of previously published studies (especially large clinical trials), would provide greater degree of inference and would yield greater patient benefit. In addition, these actions would provide better elements to the IRB and REC to evaluate future research proposals more efficiently (Harman et al., 2015).

## Role of the Institutional Review Board

The IRB and/or the REC are the check point to ensure the clinical research proposal is sound and carried out ethically. This mission requires from the reviewing process to assess whether the goals and objectives of the proposal are rigorous, the pursued outcomes present a logical clinical expectation and are methodologically achievable (MacKay, 2001). This assessment ensures the main aim of the IRB/REC to protect the participant subjects, the institution conducting the research (Guillemin et al., 2012) and warrants avoiding unnecessary patient burden (Carlisle et al., 2016). Obviously this review process relies on accurate, significant, and relevant information; the independence (absence of conflict of interests) and the integrity of its members (Harman et al., 2015; Macpherson et al., 2017) to develop a constructive decision within a reasonable uncertainty limits (Kimmelman, 2012) which leads to subject selection and the initiation of the clinical trial (Chalmers et al., 2013; Kimmelman and London, 2015).

### Embed Covariate Sex in the Review Process

As drug developers expand the clinical use of their marketed drugs based on the observations from large clinical trials, the best way to ensure the analysis of the effect of sex upon an exploratory clinical outcome is requiring its inclusion in the study protocol. In this *“bedside-to-bench-and-back-to-bedside”* approach, the study proposals need to be sufficiently justified and supported with published preclinical information to demonstrate the rationale for the new clinical study/trial application. The IRB should request a preliminary assessment of sex-divergent outcomes based on the observations that lead to the new study application. In addition all clinically relevant evidence (*“bedside”*) of prior clinical trials submitted to the IRB/REC may include a posterior evaluation of possible sex-divergent outcomes and address how the endpoints may assess the difference between male and female patients. The supporting preclinical (*“bench”*) data should include details about the sex of the cells and animals used and an analysis of sex as a covariate in cell-based assays (e.g., potency, selectivity, efflux and uptake processes, etc.) as well as animal studies (e.g., pharmacokinetic, toxicology and efficacy studies) carried out to anticipate its relevance in therapeutics (Mazure, 2016; Prager, 2017). Similarly, when a traditional *“bench-to-bedside”* approach is pursued, the IRB/REC may ensure the analysis of sex effects is evaluated along each preclinical stage without assuming a specific outcome as it has been shown that it may modify the research procedures (Maienschein et al., 2008).

The compilation of this information would allow the IRB/REC to reach a decision regarding the research proposal taking into account whether possible differences between male and female patients are addressed properly with a minimal risk of patient burden (Carlisle et al., 2016). Furthermore, the IRB should push to ensure that upcoming clinical trials are *“designed with stratified randomization by sex”* (Prager, 2017). This design may guaranty that the sex-divergent therapeutic outcomes are found or rejected based on clinical significance (Fisher and Ronald, 2010). In this context, we would encourage Regulatory Agencies and the pharmaceutical industry to incorporate this approach to bioequivalence studies, and include sufficient number of male and female subjects to ensure that possible sex differences are detected.

### Expanding the Role and Responsibility of the IRB/REC and the Pharmaceutical Industry

The IRB/REC can contribute largely to identify sex-different clinical outcomes not only with the revision and approval of the study protocol but also following up the development of the clinical trial. This would require expanding its competency and to interact with the research team or pharmaceutical company sponsoring the study on a pre-agreed schedule. This operating procedure would empower the IRB/REC to pursue a variety of actions regarding the inclusion of the assessment of covariate sex:

• First, it would ensure that the clinical trial is not stopped upon achieving a specific endpoint without sufficient clinical significance and clarity to assess the outcomes between sexes, especially when a novel indication is pursued (Henderson et al., 2015). The IRB may require scientists to evaluate and report back on a regular basis to ensure the accomplishment of the outcomes (Coleman and Bouësseau, 2008).
• Second, the IRB should push for the full publication of the aggregate results as well as categorized by sex when the sponsoring entity issues the final report. In fact, transparency of the results reports is a worthwhile target to pursue: appropriate reporting of data fosters knowledge dissemination and therefore, promotes beneficial information and prevents harmful research, or at least avoids using resources unnecessary. In this sense, it may be useful for researchers to have some kind of reporting guidelines to add to the submission of the research proposal at the review process (Nicholls et al., 2016).
• Third, the IRB, jointly with the leading scientists of the study, may foster the implementation of AD clinical trials to better determine possible endpoint differences between male and female subjects or patients (Coffey et al., 2012; Chow, 2014). Similarly, the AD clinical trials may improve the suitability of exploratory biomarkers for each sex category (Antoniou et al., 2016).
• Last, the IRB may engage and participate in the DMC, even when the scope of the DMC involves several clinical research centers disseminated in different countries. Their participation may be widened rather than limited to overall management and coordination tasks. It may assure that no information which could affect the informed consent (e.g., possible sex-divergent adverse events) is withheld, new information is provided to the scientists conducting the clinical trial as well as the integrity of the study is preserved (Chalmers et al., 2013).

The contribution of the IRBs and RECs to assess sex differences goes beyond the review process task given the nature of the findings. Their assessment *“matters scientifically, it matters ethically and it matters socially”* (Maienschein et al., 2008) as it aims to improve population health and guaranty health equality taking into account the individual (male or female) characteristics (Chapman, 2010). Although this expanding role is necessary, a review of the means available for each IRB and REC to pursue it is needed: IRBs and RECs may be ill-equipped regarding resources available, training and formation of their members, including scientific training to fully understand the research proposals (Coleman and Bouësseau, 2008; Guillemin et al., 2012). Thus, inclusion and empowering competent and knowledgeable external members in the research and practice deliberative process may help, at least initially, solving the lack of resources for an expanding role of the IRBs and RECs (Bennet and Chapman, 2010).

## Concluding Remarks

The translational perspective of covariate sex integrating preclinical and clinical evidence of TK sunitinib suggests the importance of addressing systematically sex-divergent therapeutic outcomes. This information should be available to foster and enhance future clinical research as well as to improve current treatment options. The IRBs and RECs, together with the pharmaceutical industry and other research sponsors play a key role to ensure that possible differences between male and female patients are identified, taken into account and an assessment of their clinical relevance performed. Empowering and expanding the role of IRB and REC to pursue this analysis may result in valuable inputs and improve future clinical trials outputs.

Overall, although we have focused our evidence in a specific drug used in oncology, these proposals and views may be incorporated in other therapeutic areas. Furthermore, their review could serve to echo and foster scientific discussion at the technical and ethical level to emphasize the importance of developing studies addressing sex differences that would render the possibility to personalize treatments and benefit patients.

## Author Contributions

IS conceptualized, designed the review, and wrote the manuscript, PM contributed to the conceptualization and writing of the manuscript. CF and EM contributed to the conceptualization of the manuscript. All authors participated and contributed critically to the review, discussion, and analysis of the information. All authors revised the manuscript and approved it.

## Conflict of Interest Statement

The authors declare that the research was conducted in the absence of any commercial or financial relationships that could be construed as a potential conflict of interest.

## Abbreviations

AD: adaptive design
DDI: drug–drug interaction
DMC: data monitoring committees
GIST: gastrointestinal stromal tumor
IRB: Institutional Review Board
mRCC: metastatic renal cell carcinoma
RCC: renal cell carcinoma
REC: Research Ethics Committee
TDM: therapeutic drug monitoring
TK: tyrosine kinase
